# Cost-effectiveness of Cardiac Telerehabilitation With Relapse Prevention for the Treatment of Patients With Coronary Artery Disease in the Netherlands

**DOI:** 10.1001/jamanetworkopen.2021.36652

**Published:** 2021-12-02

**Authors:** Rutger W. M. Brouwers, Esmée K. J. van der Poort, Hareld M. C. Kemps, M. Elske van den Akker-van Marle, Jos J. Kraal

**Affiliations:** 1 Vitality Center, Máxima Medical Center, Eindhoven/Veldhoven, Veldhoven, the Netherlands; 2 Department of Cardiology, Máxima Medical Center, Eindhoven/Veldhoven, Veldhoven, the Netherlands; 3 Department of Biomedical Data Sciences, Medical Decision-Making Unit, Leiden University Medical Center, Leiden, the Netherlands; 4 Department of Industrial Design, Eindhoven University of Technology, Eindhoven, the Netherlands; 5 Department of Human-Centered Design, Faculty of Industrial Design Engineering, Delft University of Technology, Delft, the Netherlands

## Abstract

**Importance:**

Cardiac telerehabilitation (CTR) has been found to be a safe and beneficial alternative to traditional center-based cardiac rehabilitation (CR) and might be associated with higher participation rates by reducing barriers to CR use. However, implementation of CTR interventions remains low, which may be owing to a lack of cost-effectiveness analyses of data from large-scale randomized clinical trials.

**Objective:**

To assess the cost-effectiveness of CTR with relapse prevention compared with center-based CR among patients with coronary artery disease.

**Design, Setting, and Participants:**

This economic evaluation performed a cost-utility analysis of data from the SmartCare-CAD (Effects of Cardiac Telerehabilitation in Patients With Coronary Artery Disease Using a Personalized Patient-Centred ICT Platform) randomized clinical trial. The cost-effectiveness and utility of 3 months of cardiac telerehabilitation followed by 9 months of relapse prevention were compared with the cost-effectiveness of traditional center-based cardiac rehabilitation. The analysis included 300 patients with stable coronary artery disease who received care at a CR center serving 2 general hospitals in the Netherlands between May 23, 2016, and July 26, 2018. All patients were entering phase 2 of outpatient CR and were followed up for 1 year (until August 14, 2019). Data were analyzed from September 21, 2020, to September 24, 2021.

**Intervention:**

After baseline measurements were obtained, participants were randomly assigned on a 1:1 ratio to receive CTR (intervention group) or center-based CR (control group) using computerized block randomization. After 6 supervised center-based training sessions, patients in the intervention group continued training at home using a heart rate monitor and accelerometer. Patients uploaded heart rate and physical activity data and discussed their progress during a weekly video consultation with their physical therapist. After 3 months, weekly coaching was concluded, and on-demand coaching was initiated for relapse prevention; patients were instructed to continue using their wearable sensors and were contacted in cases of nonadherence to the intervention or reduced exercise or physical activity volumes.

**Main Outcomes and Measures:**

Quality-adjusted life-years were assessed using the EuroQol 5-Dimension 5-Level survey (EQ-5D-5L) and the EuroQol Visual Analogue Scale (EQ-VAS), and cardiac-associated health care costs and non–health care costs were measured by health care consumption, productivity, and informal care questionnaires (the Medical Consumption Questionnaire, the Productivity Cost Questionnaire, and the Valuation of Informal Care Questionnaire) designed by the Institute for Medical Technology Assessment. Costs were converted to 2020 price levels (in euros) using the Dutch consumer price index (to convert to US dollars, euro values were multiplied by 1.142, which was the mean exchange rate in 2020).

**Results:**

Among 300 patients (266 men [88.7%]), the mean (SD) age was 60.7 (9.5) years. The quality of life among patients receiving CTR vs center-based CR was comparable during the study according to the results of both utility measures (mean difference on EQ-5D-5L: −0.004; *P* = .82; mean difference on EQ-VAS: −0.001; *P* = .92). Intervention costs were significantly higher for CTR (mean [SE], €224 [€4] [$256 ($4)]) compared with center-based CR (mean [SE], €156 [€5] [$178 ($6)]; *P* < .001); however, no difference in overall cardiac health care costs was observed between CTR (mean [SE], €4787 [€503] [$5467 ($574)] and center-based CR (mean [SE], €5507 [€659] [$6289 ($753)]; *P* = .36). From a societal perspective, CTR was associated with lower costs compared with center-based CR (mean [SE], €20 495 [€ 2751] [$23 405 ($3142)] vs €24 381 [€3613] [$27 843 ($4126)], respectively), although this difference was not statistically significant (−€3887 [−$4439]; *P* = .34).

**Conclusions and Relevance:**

In this economic evaluation, a CTR intervention with relapse prevention was likely to be cost-effective compared with center-based CR, suggesting that CTR maybe used as an alternative intervention for the treatment of patients with coronary artery disease. These results add to the evidence base in favor of CTR and may increase the implementation of CTR interventions in clinical practice.

## Key Points

**Question:**

Is cardiac telerehabilitation with relapse prevention cost-effective compared with center-based cardiac rehabilitation for the treatment of patients with coronary artery disease?

**Findings:**

In this economic evaluation of data from 300 participants with coronary artery disease enrolled in the SmartCare-CAD randomized clinical trial, patients who received cardiac telerehabilitation with relapse prevention vs traditional center-based cardiac rehabilitation experienced comparable quality of life and nonsignificantly lower cardiac-associated health care costs and non–health care costs.

**Meaning:**

This study found that cardiac telerehabilitation with relapse prevention was likely to be cost-effective compared with center-based cardiac rehabilitation and may be used as an alternative to center-based cardiac rehabilitation among patients with coronary artery disease.

## Teaser

This economic evaluation assesses the cost-effectiveness of a cardiac telerehabilitation intervention incorporating relapse prevention compared with a traditional center-based cardiac rehabilitation program among patients with coronary artery disease in the Netherlands.

## Introduction

Cardiac rehabilitation (CR) is recommended in European Society of Cardiology clinical guidelines (class IA)^1^ for the treatment of patients with coronary artery disease (CAD) because it has been associated with reductions in cardiovascular mortality and morbidity and increases in quality of life (QOL)^2,3,4^ as well as substantial decreases in health care costs.^5,6^ Moreover, CR intervention has been found to be cost-effective compared with no CR intervention, even in the modern era in which CAD is being successfully treated with optimized medical therapy and coronary revascularization.^7^ However, participation rates in current center-based CR programs remain low^8,9^ despite efforts to increase their use.^10^

Cardiac telerehabilitation (CTR) has been reported to be a safe and beneficial alternative to traditional center-based CR^11,12^; 3 small-scale studies^13,14,15^ and 2 recent systematic reviews^7,16^ found that CTR may be cost-effective compared with center-based CR. However, larger-scale studies, including comprehensive cost-effectiveness analyses, are needed.^7,16,17^

The SmartCare-CAD (Effects of Cardiac Telerehabilitation in Patients With Coronary Artery Disease Using a Personalized Patient-Centred Information and Communications Technology Platform) randomized clinical trial^18^ evaluated the effectiveness of a novel extended CTR intervention, which included video consultation and relapse prevention through on-demand coaching, compared with traditional center-based CR. The SmartCare-CAD study, which was the largest clinical trial of CTR to date, demonstrated that CTR with relapse prevention and center-based CR were equally effective, and both approaches led to sustained increases in physical activity levels, exercise capacity, and QOL after 1 year.^19^ The current economic evaluation performed a comprehensive cost-utility analysis of data from the SmartCare-CAD clinical trial. We hypothesized that the novel CTR intervention with the addition of relapse prevention that was used in the SmartCare-CAD clinical trial would be cost-effective compared with center-based CR.

## Methods

### Study Design

The SmartCare-CAD study was a prospective randomized clinical trial of patients who received CR at the Máxima Medical Center, Eindhoven/Veldhoven, which serves 2 general hospitals in the Netherlands. The trial protocol was reviewed and approved by the institutional review board of the Máxima Medical Center, and the clinical trial was registered in the Netherlands Trial Register (registration number: NL5001). All participants provided written informed consent before study entry, and the SmartCare-CAD clinical trial was conducted according to the Declaration of Helsinki.^20^ The SmartCare-CAD protocol has been described in detail elsewhere.^18^ The present economic evaluation comprised a cost-utility analysis of data from the SmartCare-CAD study, comparing the cost-effectiveness of 3 months of CTR followed by 9 months of relapse prevention with the cost-effectiveness of traditional center-based CR. This study followed the Consolidated Health Economic Evaluation Reporting Standards (CHEERS) reporting guideline for economic evaluations of health care interventions.^21^

### Study Population, Randomization, and Blinding

The study included 300 patients who received care at the Máxima Medical Center between May 23, 2016, and July 26, 2018. All patients were entering phase 2 of outpatient CR for the treatment of CAD (ie, patients with stable CAD, patients with an acute coronary syndrome, and/or patients who had received coronary revascularization). Patients were eligible if they were referred for the exercise training module of the CR program.^22^ After baseline measurements were obtained, the investigator (R. B.) randomly allocated patients on a 1:1 ratio to receive CTR (intervention group) or center-based CR (control group) using computerized block randomization. The investigator, supervising health care professionals (one of whom was H. K.), and patients were not blinded to group allocation because of the nature of the intervention. Patients were followed up for 1 year (until August 14, 2019).

### Exercise Training and Other Core Components

The exercise training module in both groups was composed according to evidence-based algorithms.^23^ Module content was determined individually and consisted of aerobic, functional, and resistance training. Other CR core components (eg, psychological counseling) occurred at the outpatient clinic for both groups.

### Control Group

Patients in the center-based CR control group participated in group-based training sessions under the supervision of physical therapists and exercise specialists (≥1 supervisor per 5 patients). Aerobic training was prescribed with a frequency aimed at two 60-minute sessions per week. Because exercise training programs were individually tailored,^23^ the number and intensity of supervised sessions varied among patients.

### Intervention Group

After allocation to the CTR intervention group, patients received access to a web-based application and were provided with a wrist-worn heart rate monitor (Mio Alpha; Physical Enterprises, Inc) and a hip-worn triaxial accelerometer (ActiGraph wGT3x-BT; Actigraph, LLC). The exercise training module began with 6 supervised group-based sessions, which were similar to those provided to the control group. Exercise training was then continued at home unless the patient preferred otherwise. Weekly video consultations with the physical therapist through the web-based application were scheduled until individual goals were achieved or the program was completed and evaluated.^23^

### Telemonitoring and Relapse Prevention

Patients in the intervention group uploaded sensor data to the web-based application at least once per week and reviewed these data during video consultations. Weekly telemonitoring guidance was concluded after 3 months; patients were then instructed to continue using their sensors and uploading data until study completion after 12 months. For this novel extension comprising on-demand coaching, the investigator evaluated sensor data every 4 weeks until study completion and notified the patient’s physical therapist when relapses occurred, which were defined as (1) nonadherence to the intervention (no data uploaded for ≥4 weeks), (2) reduced exercise training (a decrease of ≥50% in mean weekly training minutes), or (3) reduced physical activity (a decrease of ≥50% in mean time spent per day in moderate to vigorous activity). In cases of relapse, a video consultation was scheduled, in which exercise and physical activity targets and reasons for relapse were evaluated.

### Outcome Measures

#### Utilities and Quality-Adjusted Life-Years

Quality of life was assessed using 3 measures. The Dutch versions of the EuroQol 5-Dimension 5-Level survey (EQ-5D-5L) and the EuroQol Visual Analogue Scale (EQ-VAS)^24^ were administered at 3, 6, 9, and 12 months after baseline, and the Dutch version of the MacNew Heart Disease Health-Related Quality of Life Questionnaire (KVL-H)^25^ was administered at baseline and at 3 and 12 months after baseline.

Utilities were then calculated to measure patient QOL on a scale of 0 to 1, with 0 indicating worst health and 1 indicating best health. We calculated EQ-5D-5L utilities for the Netherlands using Dutch tariffs^26^ and transformed the values from the EQ-VAS to a utility scale using the following power transformation: (1) the EQ-VAS value was divided by 100, (2) the quotient was subtracted from 1, (3) the difference was then subtracted from 1, and (4) the final difference was raised to the power of 1.61.^27^ Because no EQ-5D-5L measurements were available at baseline, we converted the KVL-H scores at baseline to EQ-5D-5L utilities using mapping^28^ (eFigure 1 in the Supplement). The KVL-H utility at baseline and the utilities from the EQ-5D-5L and EQ-VAS at the other available time points were used to calculate the mean utility for each quarter of the year and for the whole year after randomization to generate the area under the utility curves, equaling quality-adjusted life-years (QALYs).

#### Costs

We estimated the societal costs for a follow-up period of 1 year; therefore, costs were not discounted. We converted costs to 2020 price levels (in euros) using the Dutch consumer price index (to convert to US dollars, euro values were multiplied by 1.142, which was the mean exchange rate in 2020).^29^ We differentiated between cardiac health care costs, noncardiac health care costs, and non–health care costs. We also distinguished between costs from CAD and CAD-associated comorbidity (classified as cardiac health care costs) and costs from other health conditions (classified as noncardiac health care costs) to isolate the impact of CTR and center-based CR for cardiac health care costs. Patients reported their medical consumption, including general health care visits, hospital visits and treatments, hospital admissions, medications received, and home care, using an adjusted version of the Medical Consumption Questionnaire from the Institute for Medical Technology Assessment.^30^ We used Dutch standard prices for health care use,^31^ supplied with mean prices for health care use from the Dutch Health Care Authority,^32^ to calculate costs. The numbers of center-based and home-based exercise training sessions were registered in case report forms. An overview and description of CTR and center-based CR intervention costs are available in eTable 1 in the Supplement.

Patients reported presenteeism and absenteeism at work using the Productivity Cost Questionnaire from the Institute for Medical Technology Assessment,^33^ and caregivers reported informal care using the Valuation of Informal Care Questionnaire from the Institute for Medical Technology Assessment,^34^ which together constituted non–health care costs. We applied a limit of 18 h/d spent on informal care to account for 6 h/d that caregivers may have spent addressing their own personal needs.^35^ The hours of absenteeism from work during the entire 1-year follow-up period were calculated using the friction cost method, which calculates productivity costs by multiplying the standard mean hourly wage by the period it takes for an organization to restore production levels to normal after an employee is absent from work because of illness. The friction period consisted of 85 days at a standard mean hourly wage of €38.09 ($43.50) per hour.^31^

### Statistical Analysis

The intention-to-treat principle was applied to all analyses. Approximately 20% of items were missing for the EQ-5D-5L, 15% were missing for the KVL-H, 15% were missing for the EQ-VAS, and 14% each were missing for the Medical Consumption Questionnaire, Productivity Cost Questionnaire, and Valuation of Informal Care Questionnaire. To reduce bias from missing data, we used multiple imputation by fully conditional specification for a total of 50 imputations. The imputation regression model contained treatment allocation and demographic and disease-specific variables. Differences in patient characteristics, costs, utilities, and QALYs between the intervention and control groups were analyzed using a 2-tailed *t* test for unequal variance at a significance level of 2-sided *P* = .05. We conducted statistical analyses using IBM SPSS Statistics, version 25.0 (IBM Corp), for Windows (Microsoft Corp). Data were analyzed from September 21, 2020, to September 24, 2021.

A cost-utility analysis is a special form of cost-effectiveness analysis in which effectiveness is expressed in QALYs. We performed the cost-utility analysis using the net benefit approach, which places a monetary value on health using willingness to pay (WTP) as a threshold for the maximum price for a QALY. An intervention is considered to be cost-effective when the net benefit (costs subtracted from the product of WTP and QALY) of the intervention is greater than the net benefit of the comparator. The base-case cost-utility analysis compared societal costs (cardiac health care costs and non–health care costs) with QALYs (based on EQ-5D-5L measurements) at 1 year. Univariate sensitivity analyses were conducted to include (1) the health care perspective (model included cardiac health care costs and QOL measured by the EQ-5D-5L), (2) utility measures from the EQ-VAS (model also included cardiac health care costs and non–health care costs measured using the friction cost method), (3) productivity costs using the human capital method (which estimated the indirect cost associated with productivity loss by measuring the value of an individual's future earnings; model also included cardiac health care costs and QOL measured by the EQ-5D-5L), and (4) all societal costs (model included cardiac health care costs, noncardiac health care costs, non–health care costs measured using the friction cost method, and QOL measured by the EQ-5D-5L).

To account for the statistical uncertainty of differences between costs and QALYs, we first applied nonparametric bootstrapping with 1000 replications. Next, we plotted cost-effectiveness acceptability curves, which represent the probability that a strategy is cost-effective as a function of WTP. At a probability of 50%, no preference for either strategy (eg, CTR vs center-based CR) exists. We then performed a value of information analysis to estimate the net gain from additional research compared with the cost of making the wrong reimbursement decision based on the current study results. Following the Wilson methods,^36^ we estimated the expected value of perfect information and the optimal sample size for a future study. We estimated fixed study costs (ie, costs of performing a future study, including personnel costs and maintenance of the ICT platform) at €350 000 ($400 000) and variable study costs (ie, mean costs of intervention and control training) at €190 per participant ($217 per participant) based on the current costs of the SmartCare-CAD clinical trial. We assumed a population of 60 000 patients in the Netherlands per year, 50% of whom were eligible for telerehabilitation, and we applied 3% discounting for a 5-year period.

## Results

**Figure 1.**
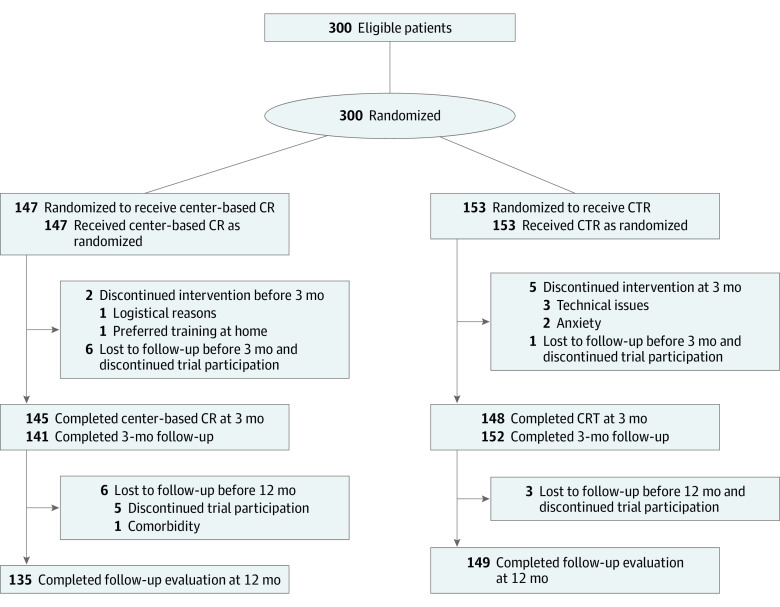
Modified CONSORT Diagram of Participants in the SmartCare-CAD Clinical Trial CR indicates cardiac rehabilitation; CTR, cardiac telerehabilitation; and SmartCare-CAD, Effects of Cardiac Telerehabilitation in Patients With Coronary Artery Disease Using a Personalized Patient-Centred ICT Platform.

Among 300 patients, 153 individuals (51.0%) received CTR (intervention group), and 147 individuals (49.0%) received center-based CR (control group). The mean (SD) age was 60.7 (9.5) years; most patients were male (266 individuals [88.7%]), currently employed (180 individuals [60.0%]), and referred for CR intervention because they had acute coronary syndrome (217 patients [72.3%]) with subsequent percutaneous coronary intervention (189 patients [63.0%]) (eTable 2 in the Supplement). A total of 16 patients were unavailable for follow-up; of those, 12 patients were in the control group (6 patients did not complete center-based CR, 11 patients discontinued participation, and 1 patient had comorbidities), and 4 patients were in the intervention group (1 patient did not complete CTR, and 4 patients discontinued participation). Patients who were unavailable vs available for follow-up were significantly younger (mean [SD] age, 56.1 [10.5] years vs 60.9 [9.4] years; *P* = .05) and had a higher median body mass index (calculated as weight in kilograms divided by height in meters squared; 30.1 [IQR, 27.2-31.0] vs 26.6 [IQR, 24.7-29.1]; *P* = .01). Other baseline characteristics did not differ between groups. A modified CONSORT diagram of study participants is available in Figure 1.

### Exercise Training and Relapse Prevention

In the first 3 months, patients in the control group attended a median of 14 supervised training sessions (range, 0-24 sessions), and patients in the intervention group attended a median of 6 supervised training sessions (range, 2-23 sessions). A total of 28 patients (18.3%) in the intervention group attended more than 6 supervised sessions, mostly because of anxiety (n = 8), chest pain (n = 5), and technical issues (n = 5).

After completion of their supervised sessions in the outpatient clinic, patients in the intervention group attended a median of 6 consultations (range, 0-11 consultations) with their physical therapist during the first 3 months. After completion of the CTR program, 98 patients (64.1%) in the intervention group attended 1 or more on-demand consultations for relapse prevention. Patients participating in on-demand consultations had a median of 1 consultation (range, 0-5 consultations), mostly because of a decrease in training intensity (39 patients [39.8%]) or nonadherence to the intervention (25 patients [25.5%]) (eFigure 2 in the Supplement).

### Utilities and QALYs

**Figure 2.**
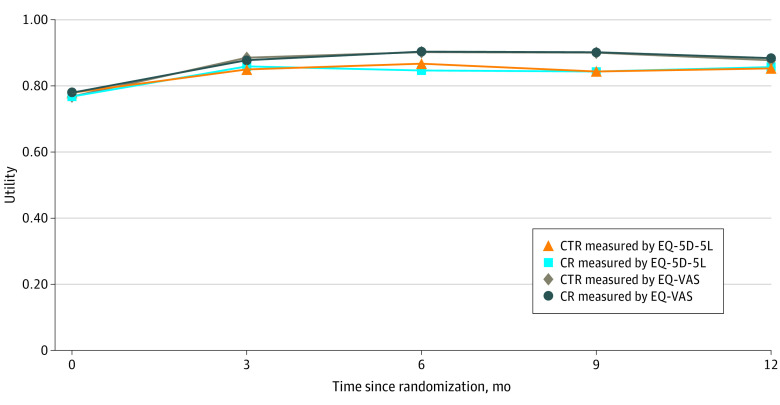
Utilities for Cardiac Telerehabilitation and Center-Based Cardiac Rehabilitation Utilities according to the Dutch versions of the EuroQol 5-Dimension 5-Level survey (EQ-5D-5L) and the EuroQol Visual Analogue Scale (EQ-VAS). CR indicates cardiac rehabilitation; CTR, cardiac telerehabilitation.

The QOL for patients receiving CTR vs center-based CR was similar throughout the study period, as measured by both the EQ-5D-5L (eg, 0.859 for CTR vs 0.850 for center-based CR at 3 months) and the EQ-VAS (eg, 0.885 for CTR vs 0.878 for center-based CR at 3 months) (Figure 2). The difference in QALYs between the CTR and center-based CR groups was not significant in any of the 4 quarters for both the EQ-5D-5L (total mean [SE] QALYs for 4 quarters, 0.841 [0.012] vs 0.844 [0.011]; difference, −0.004; *P* = .82) and the EQ-VAS (total mean [SE] QALYs for 4 quarters, 0.878 [0.008] vs 0.879 [0.008]; difference, −0.001; *P* = .92) (Table 1).

**Table 1.** Quality of Life During Cardiac Telerehabilitation and Center-Based Cardiac Rehabilitation

Measure	QALY, mean (SE)		Difference, mean	*P* value^a^
	Cardiac telerehabilitation (n = 153)	Center-based cardiac rehabilitation (n = 147)		
EQ-5D-5L (Dutch version)				
First quarter	0.814 (0.011)	0.815 (0.010)	−0.001	.94
Second quarter	0.853 (0.012)	0.859 (0.013)	−0.006	.75
Third quarter	0.845 (0.016)	0.855 (0.016)	−0.010	.63
Fourth quarter	0.851 (0.015)	0.848 (0.016)	0.002	.91
Overall	0.841 (0.012)	0.844 (0.011)	−0.004	.82
EQ-VAS				
First quarter	0.827 (0.010)	0.828 (0.009)	−0.002	.90
Second quarter	0.894 (0.009)	0.891 (0.010)	0.003	.82
Third quarter	0.901 (0.011)	0.903 (0.010)	−0.002	.89
Fourth quarter	0.889 (0.011)	0.893 (0.013)	−0.004	.82
Overall	0.878 (0.008)	0.879 (0.008)	−0.001	.92

Abbreviations: EQ-5D-5L, EuroQol 5-Dimension 5-Level survey; EQ-VAS, EuroQol Visual Analogue Scale; QALY, quality-adjusted life-year.

^a^ *P* values based on 2-tailed *t* test for unequal variance.

### Health Care Costs

The mean (SE) intervention costs per patient were €224 (€4 [$256 ($4)]) for CTR compared with €156 (€5 [$178 ($6)]) for center-based CR, representing a mean difference of €69 ($79; *P* < .001) (Table 2). The difference in intervention costs was mainly due to the costs of the sensors (€50 per participant [$57 per participant]) and the web-based application used to implement CTR (€43 per participant [$49 per participant]) (eTable 1 in the Supplement). None of the other cardiac health care costs statistically differed between groups (Table 2; eTable 3 in the Supplement). The total mean cardiac health care costs were €720 ($822) lower among patients receiving CTR (mean [SE], €4787 [€503] [$5467 ($574)]) compared with center-based CR (€5507 [€659] [$6289 ($753)]), although this difference was not statistically significant (*P* = .36).

**Table 2.** Base-Case Analysis of Mean Cardiac Health Care and Societal Costs per Patient for Cardiac Telerehabilitation and Center-Based Cardiac Rehabilitation in the First Year

Category	Cardiac telerehabilitation (n = 153)		Center-based cardiac rehabilitation (n = 147)		Difference^a^	
	Volume, %	Cost, mean (SE), € ($)^b^	Volume, %	Cost, mean (SE), € ($)^b^	Cost, mean, € ($)^b^	*P* value^c^
Cardiac health care costs						
Rehabilitation exercise training^d^	100	224 (4) (256 [4])	99	156 (5) (178 [6])	69 (79)	<.001
Physical therapy^e^	52	282 (44) (322 [50])	47	310 (53) (354 [61])	−28 (−32)	.66
Medications	98	633 (73) (723 [83])	100	667 (75) (762 [85])	−33 (−38)	.72
Cardiac care^f^	NA	1320 (327) (1507 [373])	NA	1426 (335) (1628 [382])	−106 (−121)	.79
Emergency care^g^	NA	615 (94) (702 [107])	NA	723 (130) (826 [148])	−109 (−124)	.48
Hospital stay	33	711 (141) (812 [162])	36	1141 (231) (1303 [263])	−430 (−491)	.10
General practitioner	85	194 (19) (222 [22])	93	218 (24) (249 [28])	−24 (−27)	.41
Psychological care^h^	NA	305 (56) (348 [64])	NA	364 (67) (416 [76])	−59 (−67)	.48
Paramedical care^i^	NA	149 (26) (170 [30])	NA	149 (28) (170 [31])	0	.99
Company physician	60	292 (33) (333 [37])	60	289 (35) (330 [40])	3 (3)	.95
Smoking cessation	10	34 (12) (39 [13])	15	52 (13) (59 [15])	−18 (−21)	.25
Home care	NA	28 (19) (32 [22])	NA	11 (8) (13 [9])	16 (18)	.43
Total costs	NA	4787 (503) (5467 [574])	NA	5507 (659) (6289 [753])	−720 (−822)	.36
Non–health care costs						
Absence from work (measured by FCM)	35	2711 (568) (3096 [649])	33	3319 (755) (3790 [862])	−607 (−693)	.48
Presence at work	40	2379 (523) (2717 [598])	37	2490 (537) (2844 [613])	−111 (−127)	.87
Unpaid labor	37	5249 (1170) (5994 [1336])	43	7802 (1720) (8910 [1964])	−2553 (−2916)	.19
Informal care	57	5368 (1116) (6130 [1274])	50	5263 (1159) (6010 [1324])	104 (119)	.94
Total costs (measured by FCM)	NA	15 708 (2420) (17 939 [2764])	NA	18 874 (3115) (21 554 [3557])	−3166 (−3616)	.37
Total cardiac health care and non–health care costs (measured by FCM)	NA	20 495 (2751) (23 405 [3142])	NA	24 381 (3613) (27 843 [4126])	−3887 (−4439)	.34

Abbreviations: CTR, cardiac telerehabilitation; FCM, friction cost method; NA, not applicable.

^a^ A negative cost difference indicates savings in favor of CTR.

^b^ Costs were converted to 2020 price levels (in euros) using the Dutch consumer price index. To convert to US dollars, euro values were multiplied by 1.142, the mean exchange rate in 2020.

^c^ *P* values based on 2-tailed *t* test for unequal variance.

^d^ Cardiac rehabilitation exercise training includes center-based and home-based training.

^e^ Physical therapy includes costs of physical therapy not associated with the study intervention.

^f^ Cardiac care includes cardiac outpatient visits and cardiac day treatment.

^g^ Emergency care includes ambulance transportation and emergency department visits.

^h^ Psychological care includes care by a psychologist or social worker.

^i^ Paramedical care includes occupational and speech therapy, dietician visits, and homeopathy.

### Societal Costs

Non–health care costs were largely associated with informal care (intervention group: mean [SE], €5368 [€1116] [$6130 ($1274)]; control group: mean [SE], €5263 [€1159] [$6010 ($1324)]) and unpaid labor (intervention group: mean [SE], €5249 [€1170] [$5994 ($1336)]; control group: mean [SE], €7802 [€1720] [$8910 ($1964)]), with no significant differences found between groups (informal care: mean difference, €104 [$119]; *P* = .94; unpaid labor: mean difference, −€2553 [$2916]; *P* = .19). The total non–health care costs were €3166 ($3616) lower for CTR (mean [SE], €15 708 [€2420] [$17 939 ($2764)]) compared with center-based CR (mean [SE], €18 874 [€3115] [$21 554 ($3557)]; *P* = .37). The base-case analysis, which combined cardiac health care costs with non–health care costs, revealed a nonsignificant difference in societal costs of €3887 ($4439) in favor of CTR (mean [SE], € 20 495 [€ 2751] [$23 405 ($3142)]) vs center-based CR (mean [SE], €24 381 [€3613] [$27 843 ($4126)]; *P* = .34).

### Cost-Utility Analysis

**Figure 3.**
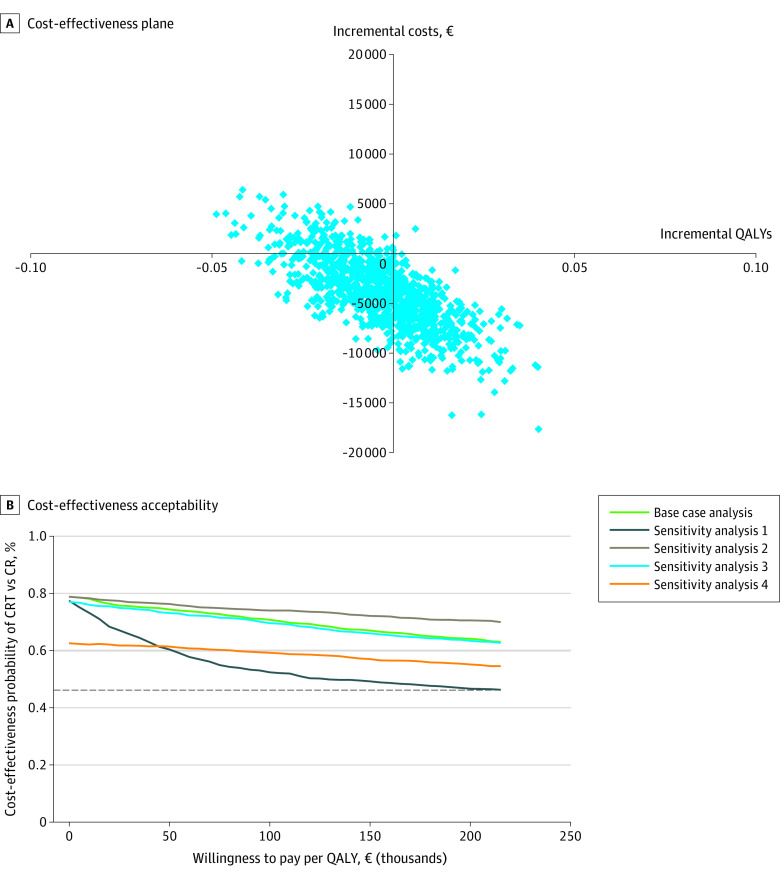
Cost-effectiveness Plane for Base-Case Analysis and Cost-effectiveness Acceptability Curves A, The cost-effectiveness plane for the base-case analysis included cardiac-associated health care costs, non–health care costs according to the friction cost method (FCM), and quality of life (QOL) measures from the EuroQol 5-Dimension 5-Level survey (EQ-5D-5L). To convert to US dollars, multiply by 1.142, the mean exchange rate in 2020 (eg, €5000 is equivalent to $5710, €10 000 is equivalent to $11 420, €15 000 is equivalent to $17 130, and €20 000 is equivalent to $22 840). B, The base-case analysis included cardiac-associated health care costs, non–health care costs according to the FCM, and QOL measures from the EQ-5D-5L. Sensitivity analysis 1 included cardiac-associated health care costs and QOL measures from the EQ-5D-5L. Sensitivity analysis 2 included cardiac-associated health care, non–health care costs according to the FCM, and utility measures from the EuroQol Visual Analogue Scale. Sensitivity analysis 3 included cardiac-associated health care costs, productivity costs according to the human capital method, and QOL measures from the EQ-5D-5L. Sensitivity analysis 4 included total health care costs, non–health care costs according to the FCM, and QOL measures from the EQ-5D-5L. The dashed horizontal line at 50% probability represents the point at which no preference for either strategy (center-based cardiac rehabilitation [CR] or cardiac telerehabilitation [CTR]) exists. To convert to US dollars, multiply by 1.142, the mean exchange rate in 2020 (eg, €50 000 is equivalent to $57 100, €100 000 is equivalent to $114 200, €150 000 is equivalent to $171 300, and €200 000 is equivalent to $228 400).

In the base-case cost-utility analysis (including cardiac health care costs and non–health care costs using the friction cost method), the incremental QALYs for CTR compared with center-based CR were spread around 0, meaning the cost-effectiveness of CTR vs center-based CR was comparable. The incremental societal costs were often lower for CTR because most data points had an incremental cost of €0 or lower (Figure 3A). Therefore, the probability that CTR was cost-effective compared with center-based CR ranged from 69% at a WTP of €0 ($0) to 86% at a WTP of €215 000 ($245 530), and the probability was 84% at a disease severity–adjusted WTP of €20 000 ($22 840) in the Netherlands.^37^ In addition, in the sensitivity analyses, the probabilities that CTR was cost-effective compared with center-based CR all remained higher than 50%, irrespective of WTP (Figure 3B). In other words, the difference in costs was mostly in favor of CTR, despite being statistically nonsignificant; therefore, CTR was likely to be cost-effective compared with center-based CR, although uncertainty remained.

The value of information analysis revealed that the optimal sample for a future randomized clinical trial would be 1600 participants per arm (eFigure 3 in the Supplement), and the total expected value of perfect information per patient would be approximately €558 ($637). This finding means that after eliminating uncertainty in future research, the expected improvement in the net monetary benefit would be €558 ($637) per patient.

## Discussion

This economic evaluation of data from the SmartCare-CAD clinical trial found that a novel CTR intervention that incorporated relapse prevention was likely to be cost-effective compared with center-based CR at a disease severity–adjusted willingness to pay of €20 000 ($22 840) in the Netherlands. In the largest CTR clinical trial to date, we observed a nonsignificant difference of €3887 ($4439) per patient in societal costs (cardiac health care costs and non–health care costs) in favor of CTR, with comparable QOL.

Our results confirm the findings of previous economic evaluations of smaller-scale CTR studies, including the FIT@Home (Home-Based Training With Telemonitoring Guidance in Low to Moderate Risk Patients Entering Cardiac Rehabilitation) clinical trial,^13^ the REMOTE-CR (Remotely Monitored Exercise-Based Cardiac Rehabilitation) study,^14^ and the Telerehab III (Effectiveness of a Comprehensive Telerehabilitation Program for the Heart) clinical trial.^15^ In the FIT@Home study, Kraal et al^13^ also found nonsignificant cost savings in favor of home-based CR for both health care and non–health care costs using a comparable intervention in a comparable study population. However, cost savings for non–health care costs, including presenteeism, were somewhat larger in the FIT@Home study (€5649 [$6451] vs €3166 [$3616] in the SmartCare-CAD clinical trial), probably owing to the use of more specific questionnaires (ie, Medical Consumption Questionnaire, Productivity Cost Questionnaire, and Valuation of Informal Care Questionnaire) in the SmartCare-CAD clinical trial, which produced a more accurate estimate. In the REMOTE-CR study,^14^ a comparable gain in QALYs was found for both groups, again in a comparable study population. However, besides intervention costs, only hospital service use and medication costs were estimated, the latter of which revealed a significant difference in favor of CTR.^14^

Compared with the Telerehab III clinical trial,^15^ the present analysis of data from the SmartCare-CAD study revealed similar cardiac health care cost savings in favor of the intervention group but no between-group differences in QALYs. In the Telerehab III study, in which CTR was provided in addition to center-based CR, QALYs increased and health care costs were lower during 2 years of follow-up (cost savings of €878 [$1003] in favor of the intervention group) compared with center-based CR alone.^15^ The main reason for these cost savings appeared to be the difference in cardiovascular rehospitalizations (32 in the intervention group vs 60 in the control group), which may also have had consequences for the QOL of participants. This difference in rehospitalizations could be associated with the fact that participants in the Telerehab III study had higher residual cardiovascular risk; thus, patients in the intervention group may have experienced greater benefit from an extended CR program. The results of the Telerehab III clinical trial suggest that prolonged CR interventions may have added value among higher-risk populations, both in terms of benefits and costs.

Our finding that CTR was likely to be cost-effective compared with center-based CR was consistent with 2 recently published systematic reviews.^7,16^ Shields et al^7^ reported that losses because of absenteeism and presenteeism were less relevant among participants receiving CR owing to their older ages (ie, approaching retirement). However, given that 60.0% of participants in the SmartCare-CAD clinical trial were still employed (and their ages were comparable with those of other CTR study populations) and that unpaid labor has substantial implications for societal costs (regardless of retirement age), the application of a societal perspective in our cost-utility analysis served as a strength, and this approach may be useful for future analyses.

Furthermore, we distinguished between cardiac and noncardiac health care costs. Although it may be difficult for patients to differentiate between health care use associated with cardiac disease vs noncardiac conditions, the high variability in noncardiac health care costs highlights the importance of separating these cost items in a comprehensive cost-effectiveness analysis.

Our findings add to the increasing body of evidence of the cost-effectiveness of CTR, suggesting that CTR is a suitable alternative to center-based CR. Yet, larger-scale analyses are still needed.^7^ The value of information analysis performed based on our study results suggested that the optimal sample for a future study would be 1600 participants per arm. Future research may support more rapid implementation of CTR interventions as part of regular care as well as the reimbursement of CTR by health care insurers and the inclusion of CTR in national guidelines.^38^ Home-based CR or CTR interventions are currently used by a small number of patients, and these interventions may be successful in increasing CR participation rates in hospitals or CR centers with limited facilities. Therefore, implementation studies are needed to assess the ways in which implementation of CTR could be facilitated in various health care settings for the treatment of different patient populations.^17^ Although CTR interventions in their current form were cost-effective compared with center-based CR, existing CTR interventions may be adapted to appeal to patients with higher residual cardiovascular risk and/or patients who are underrepresented in both center-based CR and CTR programs. These adaptations may not only enhance the implementation of CTR in regular care, but enable researchers to evaluate whether CTR is associated with increased CR participation rates.

### Limitations

This study has several limitations. First, health care consumption questionnaires are subject to recall bias. To account for potential recall bias, we calculated the intervention costs using case report forms, and we cross-checked ambiguous questionnaire responses with participants’ case report forms. Second, the current economic evaluation had a horizon of 1 year. However, because the CTR intervention occurred in the first 3 months of the study and QOL remained stable among both groups during the 9 months thereafter, we did not expect to observe between-group differences in the QOL groups after the 1-year study period. Third, the study was conducted at a single CR center serving 2 general hospitals in the Netherlands, which means that caution is warranted in the generalization of this study’s findings to other settings, especially those outside the Netherlands.

## Conclusions

The comprehensive cost-utility analysis performed in this economic evaluation found that a CTR intervention with relapse prevention was likely to be a cost-effective alternative to conventional center-based CR among patients with CAD. Together with the findings of previous economic analyses, this study’s results may be used to further support the implementation of CTR in regular practice.

## Notes

Supplement. **eTable 1.** Intervention Costs per Cardiac Telerehabilitation and Center-Based Cardiac Rehabilitation Participant **eTable 2.** Baseline Characteristics of Study Participants **eTable 3.** Mean Health Care Costs and Societal Costs per Patient During Cardiac Telerehabilitation and Center-Based Cardiac Rehabilitation **eFigure 1.** Mapping of KVL-H to EQ-5D-5L Utilities and Correction for the Dutch Setting **eFigure 2.** Median Number of Training Sessions and Video Consultations per Treatment Group **eFigure 3.** Value of Information Analysis
