# Errors in Figure 3

**DOI:** 10.1001/jamanetworkopen.2021.47432

**Published:** 2022-01-14

In the Original Investigation titled “Cost-effectiveness of Cardiac Telerehabilitation With Relapse Prevention for the Treatment of Patients With Coronary Artery Disease in the Netherlands,”^1^ published December 2, 2021, the dashed horizontal black line and colored data curves in Figure 3B were positioned incorrectly. That line and the curves should have been slightly higher in the graph, with the dashed line even with 0.5% on the y-axis and the colored data curves proportionately higher as well. This article has been corrected.^1^
