# Supplementary material for: Cost-effectiveness of Cardiac Telerehabilitation With Relapse Prevention for the Treatment of Patients With Coronary Artery Disease in the Netherlands
Source: JAMA Netw Open. 2021 Dec 2;4(12):e2136652. doi: 10.1001/jamanetworkopen.2021.36652 (PMC8640894; doi:10.1001/jamanetworkopen.2021.36652)
Supplement: Supplement. — eTable 1. Intervention Costs per Cardiac Telerehabilitation and Center-Based Cardiac Rehabilitation Participant eTable 2. Baseline Characteristics of Study Participants eTable 3. Mean Health Care Costs and Societal Costs per Patient During Cardiac Telerehabilitation and Center-Based Cardiac Rehabilitation eFigure 1. Mapping of KVL-H to EQ-5D-5L Utilities and Correction for the Dutch Setting eFigure 2. Median Number of Training Sessions and Video Consultations per Treatment Group eFigure 3. Value of Information Analysis [file jamanetwopen-e2136652-s001.pdf]

Supplementary Online Content

Brouwers RWM, van der Poort EKJ, Kemps HMC, van den Akker-van Marle ME, Kraal JJ. Cost-effectiveness of cardiac telerehabilitation with relapse prevention for the treatment of patients with coronary artery disease in the Netherlands. *JAMA Netw Open*. 2021;4(12):e2136652. doi:10.1001/jamanetworkopen.2021.36652

eTable 1. Intervention Costs per Cardiac Telerehabilitation and Center-Based Cardiac Rehabilitation Participant

eTable 2. Baseline Characteristics of Study Participants

eTable 3. Mean Health Care Costs and Societal Costs per Patient During Cardiac Telerehabilitation and Center-Based Cardiac Rehabilitation

eFigure 1. Mapping of KVL-H to EQ-5D-5L Utilities and Correction for the Dutch Setting

eFigure 2. Median Number of Training Sessions and Video Consultations per Treatment Group

eFigure 3. Value of Information Analysis

This supplementary material has been provided by the authors to give readers additional information about their work.

© 2021 Brouwers RWM et al. *JAMA Network Open*.

eTable 1. Intervention Costs per Cardiac Telerehabilitation (CTR) and Center-Based Cardiac Rehabilitation (CR) Participant

	**Total costs**		**Costs per participant**
**CTR**			
Variable costs			
Home-based training supervision (including relapse prevention)		Average duration of 13 minutes, personnel costs without overhead for one hour €39.97	€8.66[a]
Fixed costs			
Health watch	€150.00	3 year depreciation period, for 1 participant	€50.00
Hosting costs web application	€10,764	Per year for 250 participants	€43.06
Laptops (n=2)	€1836.80	5 year depreciation period, for 250 participants	€1.47
**Center-based CR**			
Variable costs			
Center-based training		Average of 10 participants in one hour training session with two physical therapists, personnel costs including overhead for one hour €57.55	€11.51[a]

[a]Training costs are presented per training session.

© 2021 Brouwers RWM et al. *JAMA Network Open*.

eTable 2. Baseline Characteristics of Study Participants

	**Centre-based CR (n=147)**	**CTR (n=153)**
Age, years*	60.5 ± 9.8	60.8 ± 9.3
Male, n (%)	132 (89.8%)	134 (87.6%)
Diagnosis, n (%)		
Stable AP	42 (28.6%)	37 (24.2%)
Unstable AP	17 (11.6%)	18 (11.8%)
NSTEMI	41 (27.9%)	41 (26.8%)
STEMI	45 (30.6%)	55 (35.9%)
Aortic valve disease (+ CAD)	2 (1.4%)	2 (1.3%)
Treatment, n (%)		
Medication	12 (8.2%)	13 (8.5%)
PCI	90 (61.2%)	99 (64.7%)
CABG	43 (29.3%)	35 (22.9%)
CABG + valve repair/replacement	2 (1.4%)	6 (3.9%)
Medication, n (%)		
Aspirin	145 (98.6%)	143 (93.5%)
P2Y12 inhibitor	105 (71.4%)	118 (77.1%)
Beta blocker	120 (88.4%)	132 (86.3%)
ACE inhibitor / ARB	105 (71.4%)	115 (75.2%)
Statin	140 (95.2%)	145 (94.8%)
VKA / NOAC	14 (9.5%)	13 (8.5%)
Risk factors, n (%)		
(Known) hypercholesterolemia	58 (39.5%)	59 (39.1%)
(Known) hypertension	62 (42.2%)	60 (39.2%)
Smoking at hospitalisation	27 (18.5%)	20 (13.2%)
Diabetes mellitus	18 (12.2%)	17 (11.1%)
Family predisposition	86 (59.3%)	86 (56.6%)
Higher education, n (%)	80 (55.2%)	70 (46.1%)
Currently employed, n (%)	89 (60.5%)	91 (59.5%)
Charlson Comorbidity Index †	1 (1)	1 (1)
BMI, kg/m^2 †	26.7 (4.9)	26.8 (4.4)
Waist circumference, cm †	102 (11)	101 (11)

© 2021 Brouwers RWM et al. *JAMA Network Open*.

Workload, % of expected*	92.1 ± 19.3	91.3 ± 18.6
Systolic blood pressure, mmHg*	123.9 ± 16.0	125.7 ± 18.6
Diastolic blood pressure, mmHg*	73.7 ± 9.0	74.8 ± 11.0
LVEF ≤ 35%, n (%)	1 (0.7%)	0 (0.0%)
Health literacy, SBSQ-D score †	5 (2)	5 (1)
Duration of hospitalisation, days †	4 (4)	4 (5)

ACE: Angiotensin-converting-enzyme; AP: angina pectoris; ARB: Angiotensin II receptor blocker; BMI: Body Mass Index; CABG: coronary artery bypass grafting; CAD: coronary artery disease; CR: cardiac rehabilitation; CTR: cardiac telerehabilitation; LVEF: left ventricular ejection fraction; NOAC: novel oral anticoagulants; NSTEMI: non-ST segment elevation myocardial infarction; PCI: percutaneous coronary intervention; SBSQ-D: Dutch version of the Set of Brief Screening Questions; STEMI: ST segment elevation myocardial infarction; VKA: vitamin K antagonist.

* Values reported as mean ± standard deviation
† Values reported as median (interquartile range).

© 2021 Brouwers RWM et al. *JAMA Network Open*.

eTable 3. Mean Health Care Costs and Societal Costs per Patient During Cardiac Telerehabilitation and Center-Based Cardiac Rehabilitation (in 2020 €)

	Telerehabilitation (n = 153)		Center-based rehabilitation (n = 147)		Difference	
	Volume, %	Costs, €	Volume, %	Costs, €	Costs, €	P[a]
Cardiac HC costs						
Cardiac rehabilitation training	100	224	99	156	69	<0.001
Physical therapy[b]	52	282	47	310	-28	0.66
Cardiac drugs	98	633	100	667	-33	0.72
Cardiac Care						
Cardiac outpatient visits	82	271	82	281	-9	0.79
Cardiac hospital treatment	44	1,036	40	1,137	-101	0.79
Treatment outside the hospital	9	13	5	8	5	0.38
Emergency care						
Emergency visits	45	337	40	406	-68	0.50
Ambulance rides	33	278	29	318	-40	0.59
Hospital stay	33	711	36	1,141	-430	0.10
General practitioner	85	194	93	218	-24	0.41
Psychological care						
Psychologist	49	270	50	334	-64	0.41
Social worker	16	35	15	30	5	0.73

© 2021 Brouwers RWM et al. *JAMA Network Open*.

eTable 3 – continued						
Paramedic care						
Occupational therapy	17	23	19	34	-11	0.42
Speech therapy	4	4	7	5	-1	0.77
Dietician	51	57	59	63	-6	0.64
Homeopathy	17	65	15	48	17	0.46
Company physician	60	292	60	289	3	0.95
Smoking cessation	10	34	15	52	-18	0.25
Home care						
Household help	1	26	1	6	20	0.32
Aid	3	1	1	5	-4	0.41
Nurse	0	0	0	0	0	
Cardiac HC costs (se)		**4,787 (503)**		**5,507 (659)**	**-720**	**0.36**
Non-cardiac HC costs						
Non-cardiac drugs	40	276	37	235	41	0.78
Non-cardiac outpatient visits	45	134	45	152	-18	0.55
Non-cardiac hospital treatment	46	11,855	45	10,861	994	0.86
Non-cardiac HC costs (se)		**12,265 (5,078)**		**11,248 (5,860)**	**1,017**	**0.86**
Total HC costs (se)		**17,052 (5,261)**		**16,755 (6,117)**	**297**	**0.96**
Non HC costs						
Absence from work (FCM)	35	2,711	33	3,319	-607	0.48
Absence from work (HCM)	35	3,056	33	3,558	-502	0.58

© 2021 Brouwers RWM et al. *JAMA Network Open*.

eTable 3 – continued						
Presenteeism	40	2,379	37	2,490	-111	0.87
Unpaid labor	37	5,249	43	7,802	-2,553	0.19
Informal care	57	5,368	50	5,263	104	0.94
Total non HC costs FCM (se)		**15,708 (2,420)**		**18,874 (3,115)**	**-3,166**	**0.37**
Total non HC costs HCM		**16,052 (2,465)**		**19,113 (3,128)**	**-3,061**	**0.39**
Total cardiac societal costs FCM (se)		**20,495 (2,751)**		**24,381 (3,613)**	**-3,887**	**0.34**
Total cardiac societal costs HCM (se)		**20,839 (2,799)**		**24,620 (3,632)**	**-3,781**	**0.36**
Total societal costs FCM (se)		**32,760 (6,275)**		**35,630 (7,618)**	**-2,870**	**0.72**

[a] T test for unequal variance. [b] Physical therapy not associated with CTR or CR. Costs are presented as means (standard errors). A negative cost difference indicates savings in favor of CTR.
HC, healthcare; se, standard error; FCM, friction cost method; HCM, human capital method.

© 2021 Brouwers RWM et al. *JAMA Network Open*.

eFigure 1. Mapping of KVL-H to EQ-5D-5L Utilities and Correction for the Dutch Setting

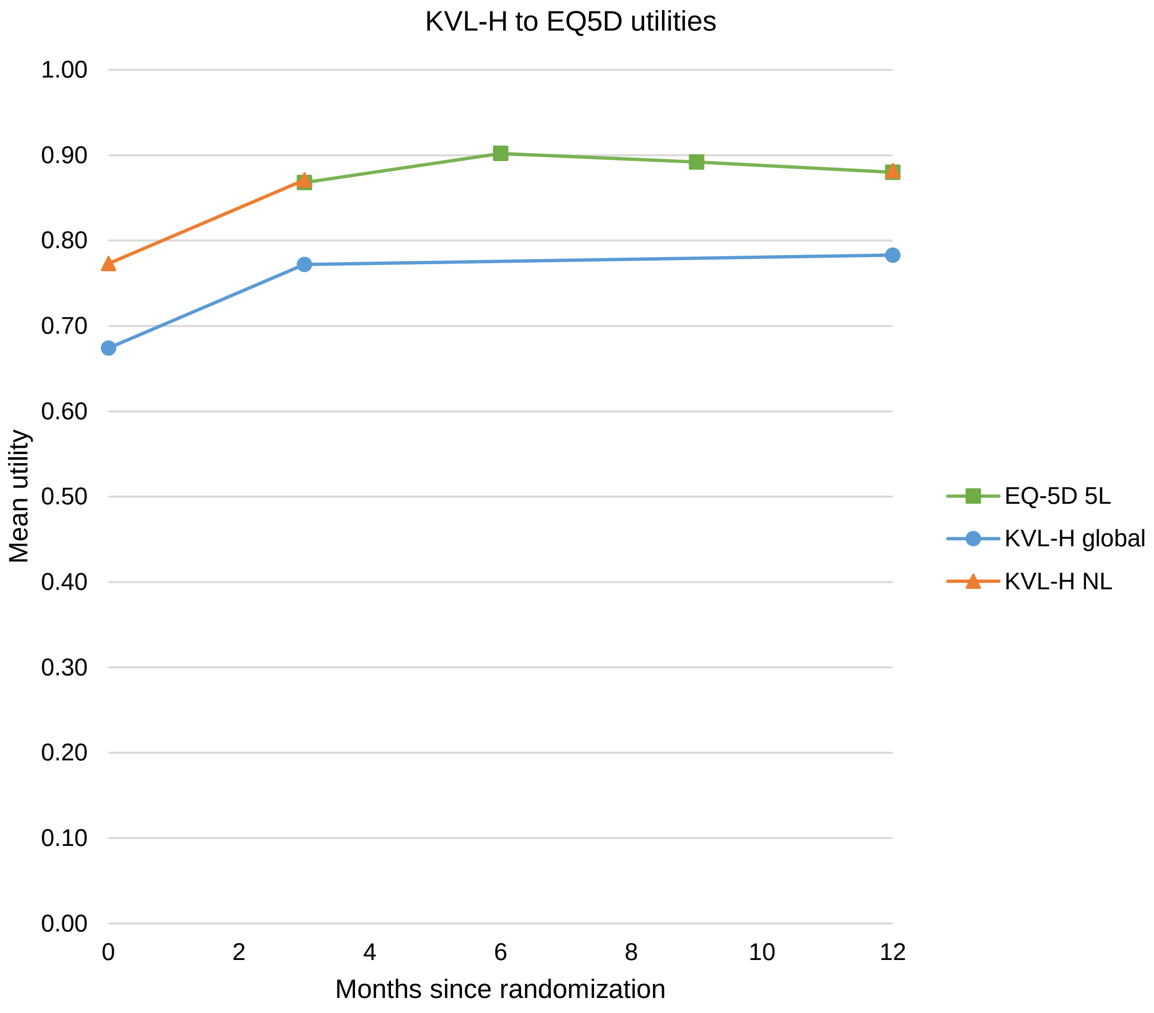

The mapping by Chen et al.[26] converts KVL-H total scores to global EQ-5D 5L utilities. We obtained a Dutch correction for the mapping by comparing the Dutch EQ5D 5L utilities and the converted global KVL-H utilities at time points where both were available (T=3 and T=12). We corrected the global KVL-H utility at baseline to a Dutch setting by addition of the mean difference between the Dutch EQ-5D 5L and global KVL-H utilities at T=3 and T=12.
KVL-H, Dutch version of the MacNew Heart Disease Quality of Life Questionnaire**;** EQ-5D 5L, EuroQol classification system.

© 2021 Brouwers RWM et al. *JAMA Network Open*.

eFigure 2. Median Number of Training Sessions and Video Consultations per Treatment Group

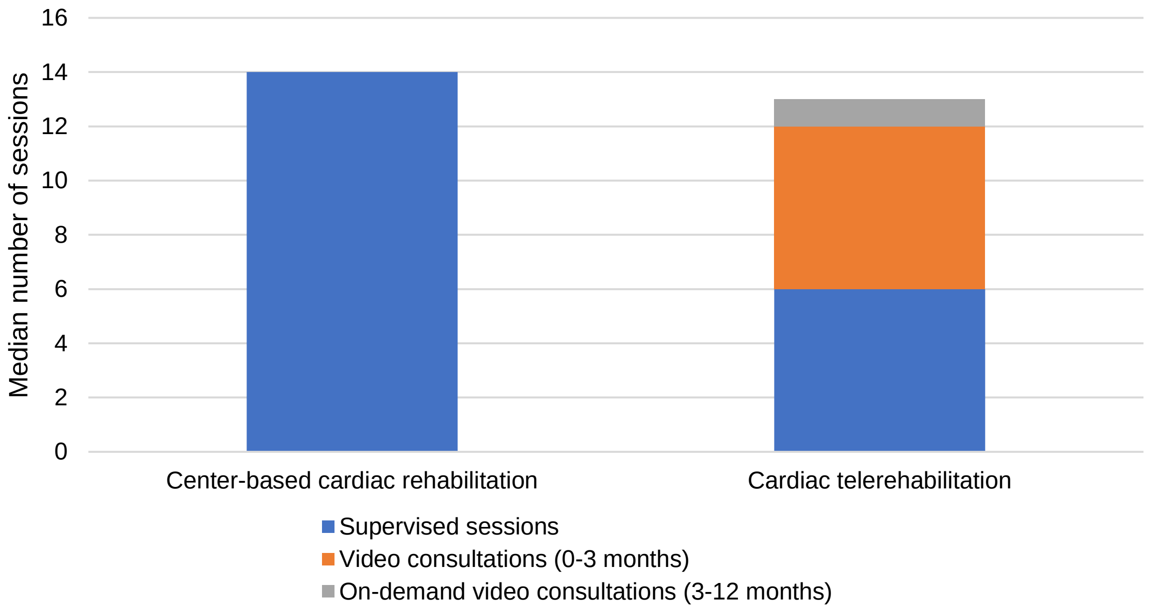

Median number of supervised training sessions in the control (center-based cardiac rehabilitation) and intervention group (cardiac telerehabilitation), and median number of (on-demand) video consultations in the intervention group.

© 2021 Brouwers RWM et al. *JAMA Network Open*.

eFigure 3. Value of Information Analysis

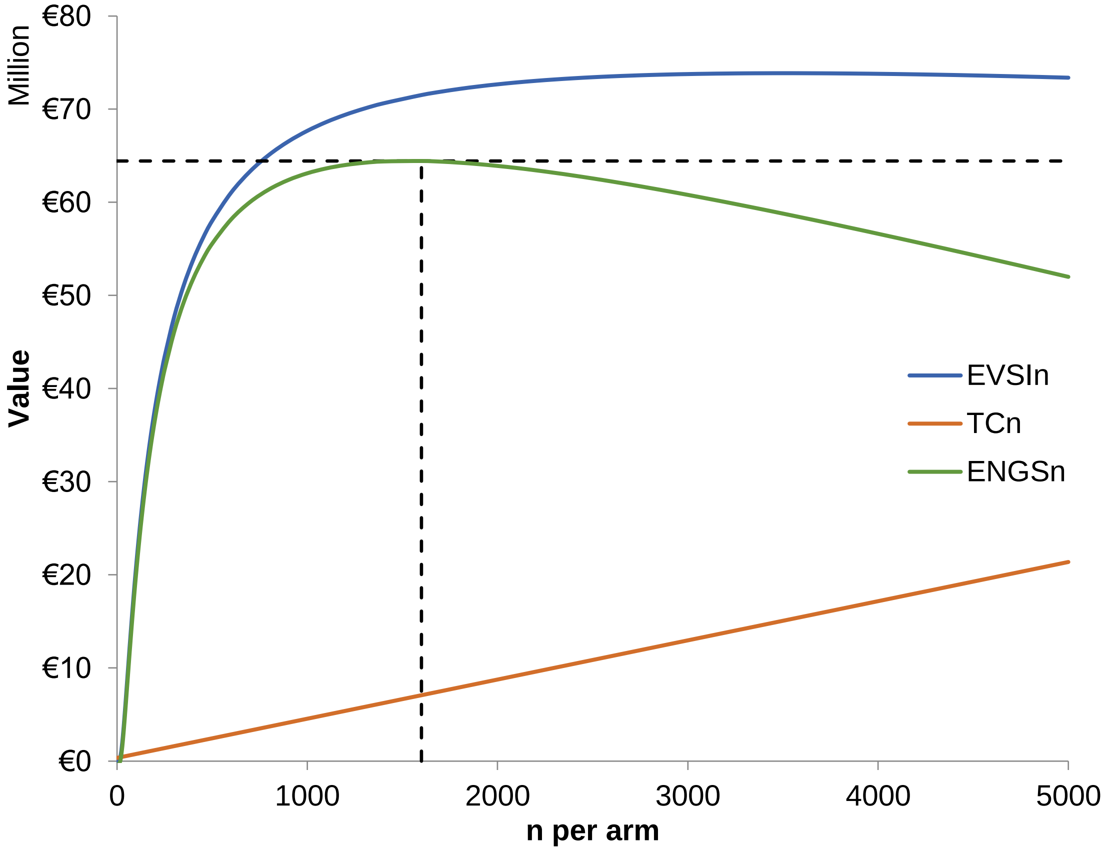

The expected value of sample information (EVSI), the total study costs (TC), and the expected net gain of sampling (ENGS) for a hypothetical future study with 2n participants. The total EVSI is an estimate of the expected benefit of a future study with a finite sample size. The ENGS is the EVSI minus the TC. The optimal sample size for a future study is reached when the ENGS is maximized.

© 2021 Brouwers RWM et al. *JAMA Network Open*.
